# Toward Standards in Clinical Microbiota Studies: Comparison of Three DNA Extraction Methods and Two Bioinformatic Pipelines

**DOI:** 10.1128/mSystems.00547-19

**Published:** 2020-02-11

**Authors:** Q. R. Ducarmon, B. V. H. Hornung, A. R. Geelen, E. J. Kuijper, R. D. Zwittink

**Affiliations:** a Center for Microbiome Analyses and Therapeutics, Department of Medical Microbiology, Leiden University Medical Center, Leiden, The Netherlands; b Experimental Bacteriology, Department of Medical Microbiology, Leiden University Medical Center, Leiden, The Netherlands; University of Trento

**Keywords:** microbiota, DNA extraction, positive controls, negative controls, bioinformatics, 16S rRNA gene amplicon sequencing, microbiome

## Abstract

When studying the microbiome using next-generation sequencing, the DNA extraction method, sequencing procedures, and bioinformatic processing are crucial to obtain reliable data. Method choice has been demonstrated to strongly affect the final biological interpretation. We assessed the performance of three DNA extraction methods and two bioinformatic pipelines for bacterial microbiota profiling through 16S rRNA gene amplicon sequencing, using positive and negative controls for DNA extraction and sequencing and eight different types of high- or low-biomass samples. Performance was evaluated based on quality control passing, DNA yield, richness, diversity, and compositional profiles. All DNA extraction methods retrieved the theoretical relative bacterial abundance with a maximum 3-fold change, although differences were seen between methods, and library preparation and sequencing induced little variation. Bioinformatic pipelines showed different results for observed richness, but diversity and compositional profiles were comparable. DNA extraction methods were successful for feces and oral swabs, and variation induced by DNA extraction methods was lower than intersubject (biological) variation. For low-biomass samples, a mixture of genera present in negative controls and sample-specific genera, possibly representing biological signal, were observed. We conclude that the tested bioinformatic pipelines perform equally, with pipeline-specific advantages and disadvantages. Two out of three extraction methods performed equally well, while one method was less accurate regarding retrieval of compositional profiles. Lastly, we again demonstrate the importance of including negative controls when analyzing low-bacterial-biomass samples.

**IMPORTANCE** Method choice throughout the workflow of a microbiome study, from sample collection to DNA extraction and sequencing procedures, can greatly affect results. This study evaluated three different DNA extraction methods and two bioinformatic pipelines by including positive and negative controls and various biological specimens. By identifying an optimal combination of DNA extraction method and bioinformatic pipeline use, we hope to contribute to increased methodological consistency in microbiota studies. Our methods were applied not only to commonly studied samples for microbiota analysis, e.g., feces, but also to more rarely studied, low-biomass samples. Microbiota composition profiles of low-biomass samples (e.g., urine and tumor biopsy specimens) were not always distinguishable from negative controls, or showed partial overlap, confirming the importance of including negative controls in microbiota studies, especially when low bacterial biomass is expected.

## Abstract (precis)

Method choice throughout the workflow of a microbiome study, from sample collection to DNA extraction and sequencing procedures, can greatly affect results. This study evaluated three different DNA extraction methods and two bioinformatic pipelines by including positive and negative controls and various biological specimens. By identifying an optimal combination of DNA extraction method and bioinformatic pipeline use, we hope to contribute to increased methodological consistency in microbiota studies. Our methods were applied not only to commonly studied samples for microbiota analysis, e.g., feces, but also to more rarely studied, low-biomass samples. Microbiota composition profiles of low-biomass samples (e.g., urine and tumor biopsy specimens) were not always distinguishable from negative controls, or showed partial overlap, confirming the importance of including negative controls in microbiota studies, especially when low bacterial biomass is expected.

## INTRODUCTION

Humans constantly interact with microbes that are present in the environment and reside on or within the human body. Recently, the attention for microbes has shifted from an exclusive interest in the pathogenicity of specific microbes toward the potential beneficial role of the microbiota in human health (1). The gastrointestinal tract contains the highest number of microbes and has been the most extensively studied body site of all human microbial communities (2). However, many other body sites are inhabited by various microbes composing a specific microbiota, such as the oral region, skin, and urogenital system. Microbial complexity varies between these niches; e.g., a healthy vaginal microbiota is often mainly composed of a few *Lactobacillus* strains, while gut and skin microbiota are usually more diverse (3).

A limiting factor in current microbiome research is that comparison of various study results is often difficult due to the application of different methodologies and lack of appropriate controls. These differences can affect data outcomes and lead to variation as large as biological differences (4). Variation can be introduced throughout the workflow, from sample collection, storage, and processing to data analysis (5–8). Recently, more attention has been devoted to standardizing the workflow of microbiome research. For instance, it was observed that DNA extraction has a large impact on obtained data (4, 9), and consensus has been achieved regarding the application of bead-beating to increase efficiency of cell wall lysis and thereby improve the yield of Gram-positive bacterial DNA (10). Nevertheless, various kits and in-house extraction methods are used across different laboratories. Recently, Costea et al. evaluated 21 DNA extraction methods across three continents and suggested one protocol, named protocol Q, as a gold standard for human fecal samples (9). They stated that it was unknown whether this method is optimal for samples other than fecal material, e.g., for low-biomass samples. To evaluate the performance of DNA extraction for low-biomass samples, it is crucial to include multiple negative controls to allow for identification of bacterial DNA introduced during the entire workflow, from sample collection to sequencing (11).

As part of optimizing the procedures for 16S rRNA gene amplicon sequencing-based microbiota studies in our facility, we evaluated three DNA extraction methods and two bioinformatic pipelines using various positive controls and negative controls. In addition, we applied these DNA extraction methods to various biological specimens.

## RESULTS AND DISCUSSION

### Mock communities pass quality control.

**FIG 1:**
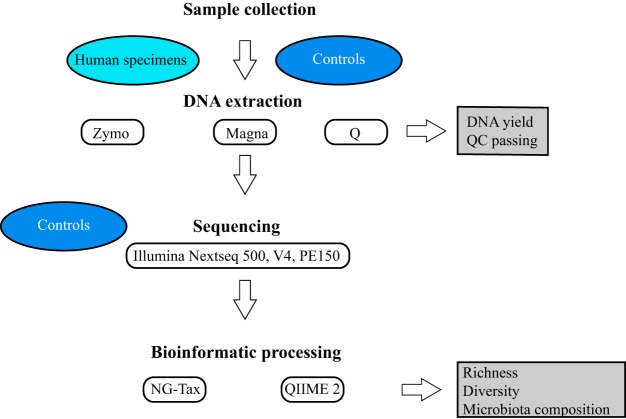
Study design workflow. DNA was extracted from human specimens and positive and negative controls using three different DNA extraction methods. DNA extraction performance was assessed on DNA yield and QC passing. Extracted DNA and positive and negative sequencing controls were sequenced. Raw sequencing data were processed using two bioinformatic pipelines. Performance was assessed on microbiota composition, richness, and diversity.

We evaluated three different DNA extraction methods and two bioinformatic pipelines for microbiota profiling through 16S rRNA gene amplicon sequencing (Fig. 1) using several positive and negative controls. Included positive controls were two bacterial mock communities (ZymoBiomics microbial community standard [here referred to as Zymo mock] and ATCC MSA2002 [here referred to as ATCC mock]) and one DNA standard. Included negative controls were DNA extraction controls and sequencing controls. Quality control (QC) passing (DNA concentration and intact genomic fragment) were evaluated to determine extraction method performance. It was expected that positive controls would pass QC, while negative controls would not. Regarding mock communities, all extractions using Zymo and Q passed QC, while for MagNA Pure 96 (here referred to as Magna) one extraction did not pass QC for both the ATCC mock community and Zymo mock community (see Table S3 in the supplemental material). This was not unexpected, as mock communities were diluted for extraction using Magna, and therefore, DNA concentrations were lower. Negative extraction controls did not pass QC for Q and Magna, but they did for Zymo. This likely represents a higher contamination load during the extraction process for Zymo, which was also reflected by higher DNA concentrations (Table S3). A full overview of all samples included in this study, their QC passing, and DNA concentrations can be found in Table S4.

10.1128/mSystems.00547-19.7 TABLE S3 Overview of all criteria and results on which extraction method and bioinformatic pipeline (combinations) are optimal. Download Table S3, XLSX file, 0.01 MB. Copyright © 2020 Ducarmon et al. 2020 Ducarmon et al. This content is distributed under the terms of the Creative Commons Attribution 4.0 International license.

10.1128/mSystems.00547-19.8 TABLE S4 Overview of all included samples, their QC passing, and DNA concentrations. Download Table S4, XLSX file, 0.01 MB. Copyright © 2020 Ducarmon et al. 2020 Ducarmon et al. This content is distributed under the terms of the Creative Commons Attribution 4.0 International license.

### Positive controls: classification, richness, diversity, and relative species abundance.

**(i) Primer choice in combination with bioinformatic pipeline choice may limit correct classification of all bacterial species in mock communities.** Performance of the three extraction methods in combination with two bioinformatic pipelines, NG-Tax and QIIME 2, was evaluated on correctly identifying richness, diversity, and relative abundances from bacterial mock communities and a DNA standard. Richness and diversity were computed at the operational taxonomic unit (OTU) level and at the genus level. Analysis of compositional profiles was performed at the genus level. Both pipelines failed to classify one organism from either mock community; NG-Tax did not detect *Cutibacterium* from the ATCC mock, while QIIME 2 did not detect *Salmonella* from the Zymo mock. The inability to detect *Cutibacterium* is most likely a combination of different internal settings and filtering steps in the computational pipelines and a primer choice issue, since the universal 515F and 806R primers are known to poorly amplify Cutibacterium acnes (12). Poor amplification of C. acnes results in limited read numbers, which may be filtered out during bioinformatic processing. These issues could likely be solved by choosing primers targeting different 16S rRNA gene regions or by using adapted V4 region primers, which do allow for accurate amplification of *Cutibacterium* (12, 13). Regarding QIIME 2 and the inability to detect *Salmonella*, there was an *Enterobacteriaceae* family with approximately expected relative abundance for *Salmonella*, and we were therefore confident this represented *Salmonella*. This *Enterobacteriaceae* family was subsequently included as *Salmonella*, and designated *Enterobacteriaceae* (*Salmonella*). This classification error likely resulted from the fact that *Enterobacteriaceae* members cannot always be discriminated based on the 16S rRNA V4 region (14).

**(ii) DNA standard and Zymo mock community data can be recovered independent of extraction protocol or pipeline.** The Zymo mock and DNA standard consist of, respectively, cell material and DNA of eight bacterial species and two fungal species. As the 16S rRNA gene was targeted, fungi should not be detected. Therefore, theoretical richness is 8 and theoretical Shannon diversity was calculated to be 2.01.

**FIG 2:**
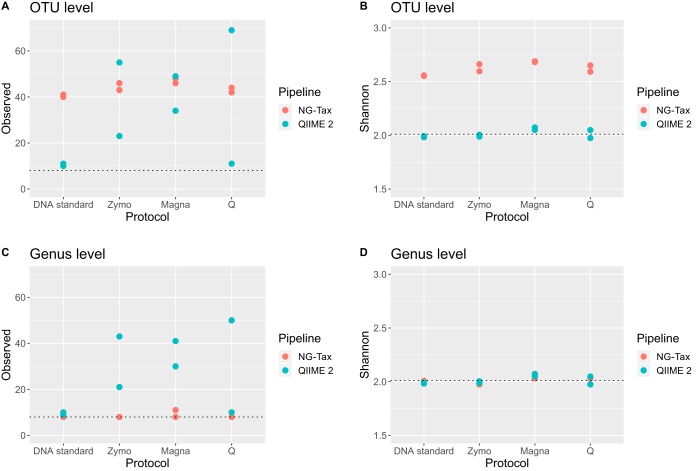
Richness (observed OTUs) and diversity (Shannon) computed for Zymo DNA and Zymo mock at the OTU level (A and B) and at the genus level (C and D) for each combination of bioinformatic pipeline and DNA extraction method. Dotted lines indicate theoretical values.

Regarding the DNA standard, NG-Tax overestimated OTU-based richness for both duplicates DNA 1 and DNA 2 (Fig. 2A; Table S3). Richness was, however, accurately retrieved at the genus level (Fig. 2C). The same was observed regarding diversity, which was overestimated at the OTU level (Fig. 2B) but accurate at the genus level (Fig. 2D). QIIME 2 approached theoretical richness and diversity values at the OTU level (Fig. 2A and B; Table S3). Richness slightly improved at the genus level (Fig. 2C), while diversity did not differ from OTU-based diversity (Fig. 2D). Thus, QIIME 2 better estimated richness and diversity at the OTU level, while NG-Tax performed better at the genus level (Table S3). This likely stems from NG-Tax finding an inflated richness due to assignment of multiple OTUs from a single organism (e.g., multiple *Enterococcus* OTUs). When OTUs are collapsed at the genus level, this is no longer a problem, probably explaining why NG-Tax can perform better at the genus level while performing worse at the OTU level.

**FIG 3:**
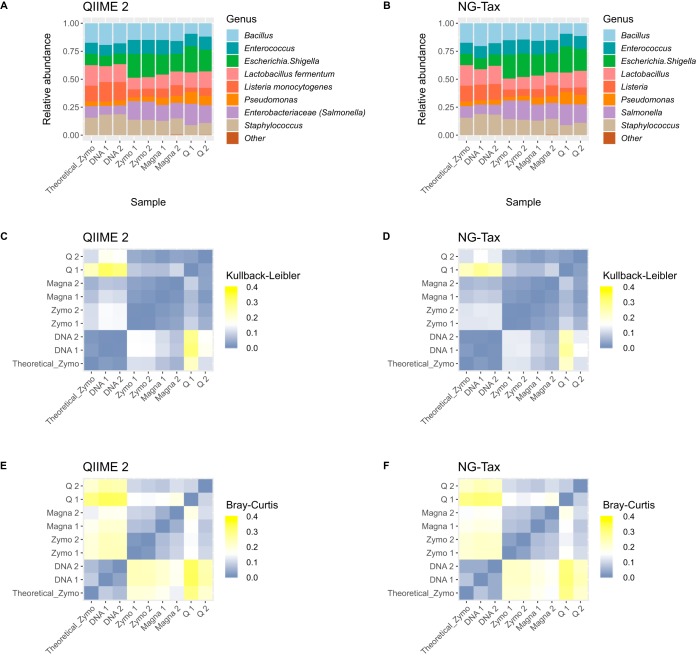
(A and B) Compositional profiles at the genus level for QIIME 2 (A) and NG-Tax (B) for Zymo mock; theoretical composition is indicated in the first bar graph. (C to F) Comparison of compositional profiles expressed by Kullback-Leibler divergence (C and D) and Bray-Curtis dissimilarity (E and F) per pipeline. QIIME 2 results are shown in panels C and E; NG-Tax results are shown in panels D and F. For both Kullback-Leibler and Bray-Curtis measures, 0 indicates an identical compositional profile, while higher numbers indicate more dissimilar profiles.

**FIG 4:**
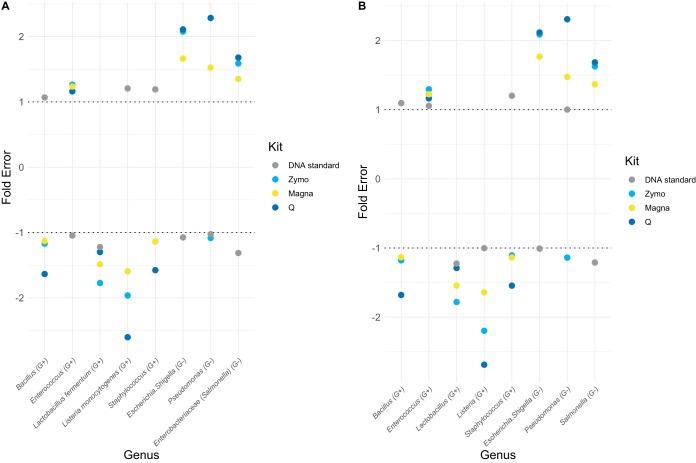
Fold error per bacterium compared to theoretical values for QIIME 2 (A) and NG-Tax (B). Genera are ordered based on being Gram positive or Gram negative. A value above 1 represents overestimation, and a value below −1 represents underestimation.

Compositional profiles of DNA 1 and DNA 2 are highly similar to theoretical abundance (Fig. 3A and B). To quantify differences in compositional profiles, Bray-Curtis dissimilarity and Kullback-Leibler divergence (Fig. 3C to F) (15) and fold errors for each taxon (Fig. 4) were determined. For the dissimilarity and divergence values, a value of zero represents an identical microbiota composition to the theoretical expectation. NG-Tax obtained values closer to zero than QIIME 2 for both DNA 1 and DNA 2, although the difference is minimal (Fig. 3; Table S2) and the performances of both pipelines can therefore be regarded as equal. A similar conclusion can be drawn from the fold errors (Fig. 4), since both pipelines accurately retrieved expected relative abundance, with all genera having a fold error between −1.5 and 1.5 (Table S3).

10.1128/mSystems.00547-19.6 TABLE S2 **C**alculation on expected 16S rRNA abundance of each bacterium in the ATCC mock. Download Table S2, XLSX file, 0.01 MB. Copyright © 2020 Ducarmon et al. 2020 Ducarmon et al. This content is distributed under the terms of the Creative Commons Attribution 4.0 International license.

Similar analyses were performed for the Zymo mock to evaluate performance of DNA extraction methods in combination with the bioinformatic pipelines. All DNA extraction methods, independent of pipeline, resulted in OTU-based richness above 20 for most samples, far higher than theoretical expectance (Fig. 2A). This is especially noteworthy for QIIME 2, as it was highly accurate in retrieving correct richness for the DNA standard, in contrast to NG-Tax. Zymo and Q protocols in combination with NG-Tax retrieved accurate genus level-based richness, while a slightly inflated richness was observed for Magna (Fig. 2C). No extraction method was consistent in retrieving correct genus level-based richness in combination with QIIME 2. Regarding diversity, all DNA extractions, independent of pipeline, retrieved highly accurate values at the genus level (Table S3). At the OTU level, however, the NG-Tax pipeline resulted in overestimation of diversity independent of the DNA extraction method and therefore the overestimation of diversity can be considered a result of bioinformatic processing. Magna extraction resulted in Bray-Curtis and Kullback-Leibler values closer to zero than Zymo and Q, independent of pipeline (Fig. 3C to F; Table S3). A similar conclusion can be drawn from the fold errors, which are lowest for Magna and pipeline independent (Fig. 4; Table S3).

Taken together, results obtained from the DNA standard indicate that QIIME 2 and NG-Tax perform equally well in general, except for overestimation of OTU level richness and diversity when using NG-Tax. Results obtained from the Zymo mock, which is a better representation of the full procedure for a microbiota study, indicate that richness is most accurate at the genus level using Zymo or Q in combination with the NG-Tax pipeline. In addition, bacterial microbiota composition profiles are best retrieved using Magna, followed by Zymo, and are pipeline independent.

In concordance with current literature (9) and independent of extraction method, a general underestimation of Gram-positive bacteria was observed, with *Enterococcus* being the sole exception (Fig. 4). This is most likely due to incomplete cell wall lysis of Gram-positive bacteria. Based on the DNA standard and the Zymo mock, we conclude that Zymo and Magna in combination with either pipeline are the best-performing combinations (Table S3). However, when high-throughput DNA extraction is required (e.g., for large cohort studies), Magna may be preferred from a practical point of view, although it overestimates richness independent of pipeline.

In general, overestimation of OTUs may stem from the 100% identity setting for clustering, combined with the natural divergence of the 16S rRNA gene (16, 17). There is no current consensus on OTU identity setting, and cutoffs between 97% and 100% are most commonly used (18). An advantage of the 100% cutoff is that unique taxa differing a single nucleotide are clustered into different OTUs. A disadvantage is that as intragenomic diversity in the 16S rRNA gene is common within bacterial genomes, a 100% cutoff can lead to multiple OTUs stemming from a single bacterium and thereby inflate richness (17). In addition, using a 100% cutoff can theoretically inflate richness due to sequencing errors and requires computational denoising. Apart from biological explanations, the different algorithms and internal filtering steps used in QIIME 2 and NG-Tax can affect the outcome for richness.

**(iii) ATCC mock is recovered incorrectly, independent of extraction protocol or pipeline.** The ATCC mock consists of 20 unique bacterial species, with four of them belonging to two genera (*Staphylococcus* and *Streptococcus*). Therefore, theoretical richness at the OTU level would be 20, but it would be 18 at the genus level. In addition, these 20 unique bacterial species come from different environments, including gut, oral, and skin microbiota.

**FIG 5:**
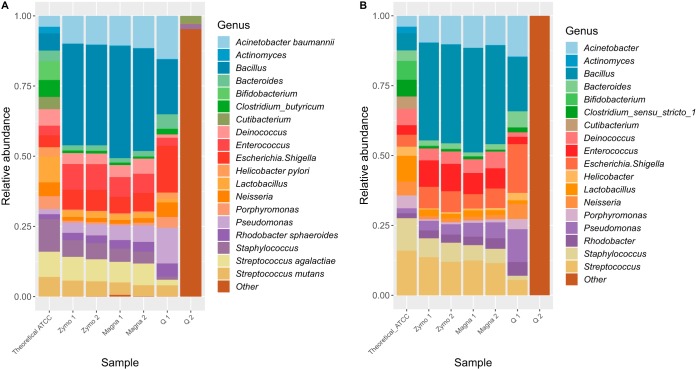
Compositional profiles at genus level for QIIME 2 (A) and NG-Tax (B) for the ATCC mock. Genus “*Other*” is the sum of the relative abundance of all genera not listed in the color key.

No values close to the theoretical profiles for the ATCC mock for any extraction method/bioinformatic pipeline were observed, and one sample from Q consisted almost entirely of nonclassifiable reads (Fig. 5), indicating sample-related issues. *Bacillus* was highly overrepresented in all other samples, with a relative abundance of >30% in Zymo- and Magna-extracted samples, while 6.13% is expected. Curiously, after the first mechanical lysis step in Q, we could culture Bacillus cereus and Cutibacterium acnes (identification scores of 1.90 and 2.00, respectively), as well as Bacillus cereus (identification score 2.05) after mechanical lysis in Zymo. This is clinically important, as it means that infectious materials cannot be considered safe or noninfectious after mechanical lysis. As culturing of B. cereus indicates that cell wall lysis was incomplete, it would be expected that its relative abundance was underestimated, contrary to what was observed. Another research group recently reported a similar overrepresentation of *Bacillus* in the ATCC community (19). The ATCC itself was also unable to retrieve abundances close to theoretical expectation, either with 16S rRNA gene amplicon sequencing or with shotgun sequencing (20). Several reasons could explain this discrepancy between theoretical profiles and obtained profiles. For example, physical cell-to-cell interactions or the presence of different metabolites may interfere with DNA extraction (16, 21). Therefore, based on this synthetic community, no conclusions on the optimal extraction-pipeline combination could be made. This proposed positive control prompts the question of whether mock communities are always reliable for assessing performance of DNA extraction methods. As can be observed from the Zymo mock, DNA extraction kits do not necessarily inflict observed deviations but may rather be a result of mock community-specific properties. Outcomes may depend on extraction kit-community type combination, indicating the potential necessity to use a positive control that strongly resembles the investigated microbiome.

### Negative controls: inconsistently contaminated.

Negative controls were taken along for each extraction method to check for kit-specific contaminants, which are especially relevant for deciding whether low-biomass samples contain real microbiota. Regarding Zymo, clear kit contaminants were *Pseudomonas* and *Delftia* (Fig. S2A and C), consistent across the different pipelines at the genus level and with previous findings (11, 22). For Magna and Q, specific contaminants were less obvious, although *Pseudomonas* was present. Generally, negative controls mostly consisted of genera commonly found in gut and oral microbiota, most of them also previously described as contaminants (11). In addition, negative sequencing controls were taken along, and in this case no consistent contaminants could be observed (Fig. S2B and D). Potential contamination sources are multifold, such as kit contamination, index hopping, or well-to-well contamination (23, 24). Index hopping is, however, not a likely source of contamination, as the negative control for Magna was sequenced in different lanes, and profiles look highly similar (Fig. S2A and C). Additionally, we did not observe index hopping in our positive controls.

10.1128/mSystems.00547-19.3 FIG S2 Relative abundance plots for DNA extraction negative controls (A and C) and sequencing controls (B and D) for both bioinformatic pipelines. Genus “*Other*” is the sum of the relative abundance of all genera not listed in the legend, while “*Not_Available*” indicates the proportion of reads which could not be classified. Download FIG S2, EPS file, 0.8 MB. Copyright © 2020 Ducarmon et al. 2020 Ducarmon et al. This content is distributed under the terms of the Creative Commons Attribution 4.0 International license.

One of the contaminants we identified has not been previously described as a contaminant, namely, *Clostridioides*. This likely represents Clostridioides difficile, and contamination by this bacterium can be explained by the fact that DNA extractions were performed in our National Reference Laboratory for C. difficile, which probably contains minor amounts of C. difficile spores at most time points. C. difficile contamination on laboratory surfaces has also recently been described for another clinical microbiology laboratory (25).

By incorporating this information with the Zymo positive controls, it can be concluded that Zymo and Magna are most optimal. Magna most accurately captured the expected community profile, while kit-specific contaminants are clear and easy to discriminate from biological signal using Zymo (Table S2). When investigating different biological sample types, it would be ideal to use a kit for which kit contaminants do not overlap the biological signal, e.g., *Pseudomonas* contamination when studying sputum samples from cystic fibrosis patients, who are frequently colonized with *Pseudomonas* spp. However, this would require contaminants to be stable across batches, which has been shown to not be the case (22).

### Automatic Magna extraction yields the lowest DNA concentrations for biological samples.

Twenty-seven biological samples were available per extraction protocol (Table S1), and Q was most successful in passing QC (22/27), followed by Zymo (20/27) and Magna (17/27) (Table S3), although differences were not statistically significant (Cochran’s Q test, *P* = 0.178). QC passing was based on DNA concentration and intact genomic fragments. DNA concentrations were, on average, lowest for Magna, while yields were comparable between Q and Zymo (Fig. S1). Processing of raw sequencing data from biological samples was performed using the NG-Tax pipeline at the genus level.

10.1128/mSystems.00547-19.2 FIG S1 Genomic DNA concentrations per extraction method for each biological specimen. VSCC, vulvar squamous cell carcinoma. Download FIG S1, EPS file, 1.7 MB. Copyright © 2020 Ducarmon et al. 2020 Ducarmon et al. This content is distributed under the terms of the Creative Commons Attribution 4.0 International license.

10.1128/mSystems.00547-19.5 TABLE S1 Overview of collection procedures and processing of the different biological samples. Download Table S1, XLSX file, 0.01 MB. Copyright © 2020 Ducarmon et al. 2020 Ducarmon et al. This content is distributed under the terms of the Creative Commons Attribution 4.0 International license.

### Fecal microbiota analysis is only slightly affected by the applied DNA extraction methods.

**FIG 6:**
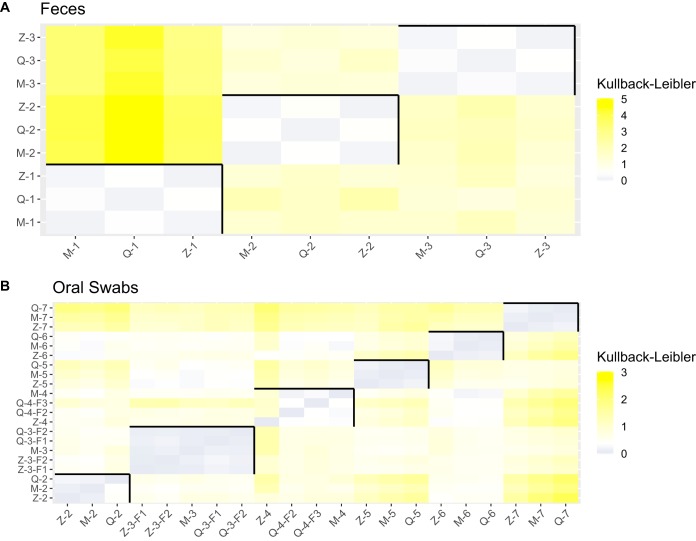
Kullback-Leibler divergence heat maps of feces (A) and oral swabs (B). Black lines group unique biological samples. Gray indicates highly similar composition, while yellow indicates divergence in composition. F1, F2, and F3 represent samples which have been sequenced in duplicate but on different flow cells.

DNA extracted from fecal samples using the three different protocols all passed QC. Magna, Zymo, and Q achieved average concentrations of approximately 29 ng/μl, 111 ng/μl, and 212 ng/μl, respectively (Fig. S1). While DNA yields varied between extraction methods, all were sufficient for sequencing. Microbiota profiles were comparable between extraction methods for each sample (Fig. S3A). In addition, differences in compositional profiles were quantified using Kullback-Leibler divergence (Fig. 6A). The heat map in Fig. 6A shows that technical variation induced by DNA extraction method is much lower than biological variation between feces samples. Profiles of the feces donors contained many bacterial genera commonly present in fecal microbiomes (26, 27). Healthy fecal microbiomes largely consist of the phyla *Bacteroidetes* and *Firmicutes* (∼90%), while *Actinobacteria* and *Proteobacteria* are present in smaller proportions. At the genus level, *Bacteroides*, *Prevotella*, and *Faecalibacterium* are among the most prevalent genera (3), all of which were found in high abundance in this study.

10.1128/mSystems.00547-19.4 FIG S3 Relative abundance plots for feces (A), oral swabs (B), urine (C), saliva (D), colorectal cancer tissue (E), colorectal cancer supernatant (F), VSCC tissue (G), and formalin-fixed VSCC (H). Sample labels are constructed in the following way: extraction protocol-sample number-flow cell number (in case of in-duplicate sequencing of the same DNA). Genus “*Other*” is the sum of the relative abundance of all genera not listed in the legend, while “*Not_Available*” indicates the proportion of reads which could not be classified. Download FIG S3, PDF file, 0.1 MB. Copyright © 2020 Ducarmon et al. 2020 Ducarmon et al. This content is distributed under the terms of the Creative Commons Attribution 4.0 International license.

### Microbiota profiles of oral swabs are consistent, despite low DNA yields.

Out of 18 DNA extractions, 15 passed QC for oral swabs. Only for Zymo did all extractions pass QC. DNA yields were highly variable for all extraction methods, ranging from 0.12 to 6.34 ng/μl. Half of the extractions (9/18) yielded a concentration below 1 ng/μl. All compositional profiles were dominated by *Streptococcus*, *Prevotella*, *Haemophilus*, and *Veillonella*, which was individual independent. In addition, technical variation induced by DNA extraction and subsequent steps was lower than biological variation (Fig. 6B). The oral microbiota, like the gut microbiota, is highly diverse. Nevertheless, a certain core of genera (e.g., *Streptococcus* and *Prevotella*) is present in most people, all of which were found in our study (3, 28, 29). Together, the good QC passing rate, DNA concentrations, and consistency of compositional profiles between extraction methods lead us to conclude that all three methods work well for oral swabs.

### Applied methodology yields inconsistent results for the urine microbiota.

During the last decade, microbiota studies showed that urine contains a bacterial microbiota (30, 31). Despite using 30 to 40 ml of urine and centrifugation prior to extraction (32), we were not able to convincingly capture a urinary microbiota for all samples (Fig. S3C). DNA concentrations were high for an infected sample (between 13 and 42 ng/μl), but concentrations for the other samples were between 0.11 and 0.99 ng/μl. Six out of nine samples passed QC. For the infected sample with a high bacterial load, we were able to classify the cause of infection to *Enterobacteriaceae*, which is in agreement with the fact that most urinary tract infections (UTIs) are caused by members of the *Enterobacteriaceae*. One urine sample showed high similarity to negative controls for respective kits, with nonclassifiable reads for Q and Magna, and high relative abundance of *Pseudomonas* for Zymo (Fig. S3C). Another urine sample contained a high *Lactobacillus* relative abundance, which has previously been shown to be prevalent in urine samples (31). *Lactobacillus* could be cultured in 15% of urine samples collected by a transurethral catheter and was thereby the most prevalent genus cultured (31). Another small-scale study found that for five out of six patients, *Lactobacillus* was detected in midstream urine samples and its relative abundance was between 22 and 80% (30). In addition, the presence of *Atopobium*, *Gardnerella*, *Prevotella*, and *Anaerococcus* points toward an existing urinary microbiota (33). However, *Pseudomonas*, a common Zymo kit contaminant, was still found in this urine sample, and for Magna more than 25% of reads could not be classified (Fig. S3C). This could indicate that the biological signal is not much stronger than contamination, and therefore, a mixed profile is observed. Further efforts and method optimization should be undertaken, although this can be difficult to implement in routine work (34). In addition, culturing could be used as a follow-up method to confirm that contaminants are not viable bacteria but rather bacterial DNA.

### Saliva samples with long storage time and multiple freezing-thawing cycles seem unsuitable for microbiota research.

DNA yield from included saliva samples was lower than described in the literature (35, 36) (Fig. S1). Only a single DNA extraction had a concentration of slightly above 1 ng/μl (1.18 [Table S4]), while all other extractions had concentrations between 0.04 and 0.68 ng/μl. This is most likely associated with storage duration (∼15 years) and the fact that samples were thawed and refrozen several times. This also explains why only three out of nine DNA extractions passed QC. The included saliva samples were chosen because investigators within our facility were interested to see if microbiota studies could be performed using these samples. Compositional profiles consisted of a mixture of genera present in the normal oral microbiota (*Oribacterium*, *Prevotella_7*, *Prevotella_9*, and *Streptococcus*) (3), genera present in our negative controls (*Pseudomonas* and *Delftia*), and nonclassifiable reads (Fig. S3D). In combination with low DNA yields, it is likely that a mixture between biological signal and contamination signal is present. Therefore, we consider the applied extraction methods unsuitable for saliva samples with a long duration of storage and multiple freezing-thawing cycles.

### The colorectal cancer microbiota present in biopsy specimens was indistinguishable from negative controls or fecal microbiota.

As colorectal cancer development has been associated with specific gut bacteria, we were interested to see if colorectal cancer tissue itself also contained bacteria (37, 38). DNA concentrations were sufficient for all samples to pass QC, but extracted DNA was likely mostly human derived. Two of three extraction methods were not successful, as samples extracted using Zymo and Magna showed high similarity to their respective negative controls (Fig. S3E). Using Q, *Bacteroides*, *Fusobacterium*, and *Gemella* were identified, all previously associated with colorectal cancer development (37, 39). Several gut commensals, including *Faecalibacterium* and *Escherichia-Shigella*, were present in both the negative controls and these colorectal cancer samples. It is therefore difficult to discriminate whether these are contaminant bacteria or whether they represent biological signal.

We hypothesized that if the material was spun down, the supernatant would contain more bacteria than the cancer tissue. DNA concentrations of supernatant were between 0.16 and 2.32 ng/μl, and seven out of nine DNA extractions passed QC (Table S4). For one sample, it was clear that across all methods, many genera were observed which were present in negative controls (e.g., *Pseudomonas*), or reads could not be classified at all (Fig. S3F). A second sample seemed to contain a real microbiota. Profiles were consistent across extraction methods, did not contain many contaminants, and had specific bacteria previously linked to colorectal cancer (e.g., *Fusobacterium*) (37). The third sample showed a profile reflecting a mix between biological signal and technical contamination. Profiles were consistent across methods and contained genera representative of a gut microbiota, but they also contained nonclassifiable reads and contamination. Therefore, profiles are likely a mixture of biological signal and technical contamination, and further optimization is necessary prior to using this sample type for experimental studies. We have the same recommendation for colorectal cancer sample types as for urine, as discussed above.

### It remains unclear whether HPV-negative vulvar squamous cell carcinoma biopsy specimens contain a bacterial microbiota.

Vulvar squamous cell carcinoma (VSCC) has different etiological pathways, of which one is associated with human papillomavirus (HPV). The counterpart is nonvirally related and is frequently associated with lichen sclerosis, a benign chronic inflammatory lesion, and *TP53* mutations (40, 41). We extracted DNA from HPV-negative VSCC tissue as a pilot study to determine if investigating the relationship between bacterial microbiota and HPV-negative VSCC would be potentially feasible. DNA concentrations were high (Fig. S1), only for three extractions below 1 ng/μl, and eight out of nine extractions passed QC. However, DNA was probably again largely human derived. This was reflected in the obtained microbiota profiles, as most reads were not classified or the profiles showed high similarity to negative controls (e.g., high abundance of *Pseudomonas*) (Fig. S3G). Therefore, it is unlikely that this cancer tissue contains bacteria, or bacteria are so lowly abundant that they are overshadowed by contamination load. In general, the vulvar microbiota has not been extensively studied. A recent study on vulvar microbiota observed that *Lactobacillus*, *Corynebacterium*, *Finegoldia*, *Staphylococcus*, and *Anaerococcus* are most abundant on this body site, but the use of negative controls was not reported (42). These genera are also part of the vaginal microbiota and might be sampling contamination or reflect high similarity between vulvar and vaginal microbiota.

A large amount of formalin-fixed VSCC materials are stored in a biobank at our facility. To investigate whether this sample collection could be used for microbiota profiling, DNA was extracted from three formalin-fixed VSCC samples. DNA concentrations were all below 0.3 ng/μl, and only two out of nine extractions passed QC (Table S4). One sample extracted with Q was excluded from further analysis, as no reads were present after sequencing. Extraction and sequencing of formalin-fixed material pose additional problems, as DNA molecules could be highly fragmented and too short for amplicon sequencing of the V4 region (43). For Zymo, samples resembled negative controls, with *Delftia* and *Pseudomonas* being highly abundant (Fig. S3H). The same samples showed completely different microbiota profiles when using protocol Q or Magna. Both extraction methods showed genera commonly found in the lower urogenital tract, including *Streptococcus*, *Prevotella*, and *Gordonia* (3, 27). However, many of these genera were also detected in negative controls. On the basis of these findings in combination with low DNA yield and inconsistent profiles across extraction methods, we conclude that no reliable bacterial microbiota profile could be identified in these samples. For both VSCC types, we suggest the same way forward as for urine samples.

### Sample groups with and without biological signal cluster apart.

**FIG 7:**
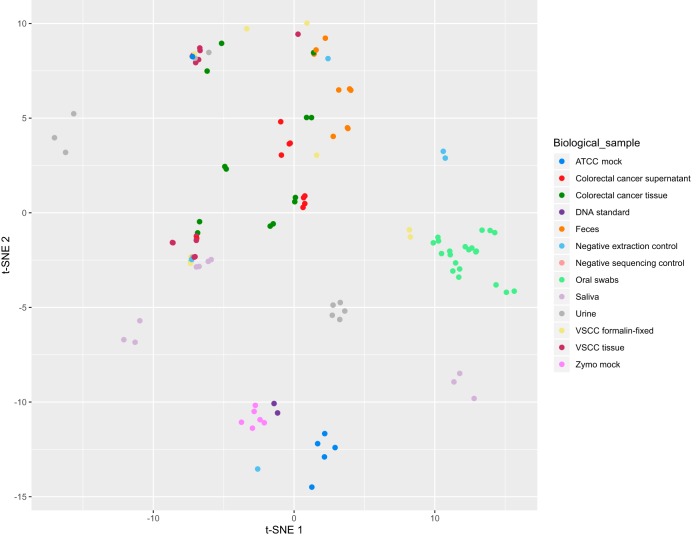
Bray-Curtis distance measures visualized by t-distributed stochastic neighbor embedding (t-SNE) for all samples. Each dot in the plot represents a single sample, and short distances between samples indicate high similarity.

Lastly, we performed t-distributed stochastic neighbor embedding (t-SNE) clustering using Bray-Curtis measures on all samples used in the present study (Fig. 7) (44). Based on microbiota composition as measured by Bray-Curtis, t-SNE projects points in a two-dimensional space while maintaining local structures present in high-dimensional space. Clear clusters could be identified for Zymo positive controls, feces, oral swabs, and ATCC mock (all but one sample) (Fig. 7). Other biological samples and negative controls were more dispersed throughout the plot, indicating that either more biological or technical variation was present. This is in agreement with our detailed analysis, showing that their microbiota cannot necessarily be distinguished from the negative controls. This highlights the importance of including negative controls in microbiota studies, which has previously been shown in two studies aiming to unravel the placental microbiota (45, 46) and is increasingly recognized in the field. It is currently unclear whether a placental microbiota exists, but when comparing placental samples of healthy deliveries to included negative controls, microbiota compositions could not be distinguished (45, 46).

### Strengths and limitations.

The current study had several strengths and limitations. By using a positive control of cell material with a corresponding DNA standard, we differentiated variation induced from sequencing procedures and DNA extraction. We demonstrate the importance of using positive and negative controls in microbiota studies, and show that negative controls are crucial for interpretation of low-biomass samples. Another strength of the study was that for several higher-biomass biological samples (feces and oral swabs), we showed that technical variation was much smaller than biological variation. A shortcoming of the study is that we did not perform any other quantification next to 16S rRNA gene sequencing (e.g., quantitative PCR [qPCR]), which may be particularly useful for quality control of the ATCC mock. Furthermore, the current study used only three unique samples of most biological sample types. Especially for samples for which DNA extraction was challenging (urine samples and colorectal cancer supernatant), a higher number of unique samples would have allowed for a more thorough evaluation.

### Conclusion.

The current study evaluated three DNA extraction methods and two bioinformatic pipelines for bacterial microbiota profiling using several positive and negative controls and a range of biological specimens. All three extraction methods quite accurately retrieved theoretical abundance of the Zymo mock but not of the ATCC mock. For DNA extraction, we recommend using the Zymo and Magna protocols, since they showed good overall performance for all samples. The sequencing procedure induced only minor variation, as shown using a DNA standard. We furthermore showed that the NG-Tax and QIIME 2 pipelines perform equally well overall, each having their specific flaws.

By including negative controls and comparing these with low-biomass samples, we evaluated whether low-biomass samples consisted of technical noise, biological signal, or a mixture. In most cases, identification of a unique microbiota was not achieved, highlighting the importance of negative controls and sufficiently sensitive methods. The results from this study can help other microbiome study groups to select an appropriate DNA extraction method and bioinformatic pipeline. Lastly, we hope this study contributes to further awareness of the usage of controls, especially when studying low-biomass samples.

## MATERIALS AND METHODS

### Sample collection and preprocessing.

Eight different biological specimens were included in this study, namely, feces, urine, saliva, oral swabs, colorectal cancer tissue, colorectal cancer supernatant, vulvar squamous cell carcinoma tissue, and formalin-fixed vulvar squamous cell carcinoma. For each biological specimen, three unique samples were included. Only for oral swabs, six unique samples were included (Table S1). These samples were anonymized and treated according to the medical ethical guidelines described in the *Code of Conduct for Proper Secondary Use of Human Tissue* of the Dutch Federation of Biomedical Scientific Societies (https://www.federa.org/). A detailed overview of sample types, sample processing and storage conditions can be found in Table S1.

### Mock communities and DNA standard.

Two mock communities (ZymoBiomics microbial community standard [Zymo Research, Irvine, CA] and 20-strain even-mix whole-cell material [ATCC MSA2002; ATCC, Wesel, Germany]) were included as positive controls for DNA extraction. The exact composition and relative abundances of 16S rRNA gene copies were provided on the product sheet for the ZymoBiomics microbial community standard (here referred to as Zymo mock), while for ATCC MSA2002 (here referred to as ATCC mock), we calculated expected 16S rRNA gene profiles based on genomic information (Table S2). The ZymoBiomics microbial community DNA standard (here referred to as DNA standard) was taken along as a positive sequencing control.

### DNA extraction procedures.

Cancer samples were preprocessed for DNA extraction comparably to a recent study on pancreatic cancer microbiota (48), urine samples according to a recent publication on how to study urinary microbiota (32), and other samples according to in-house methods for sample processing (Table S1). For solid cancer samples, the beating steps during preprocessing were performed using a Qiagen TissueLyser LT (Qiagen Benelux, Venlo, The Netherlands) at 50 Hz for 1 min (Table S1). As single saliva samples did not contain sufficient volume for multiple extractions, several samples from the same individual were pooled to obtain the appropriate volume. DNA was extracted in duplicate from three unique samples for each biological material, only for oral swabs from six unique samples, and from the two mock communities. DNA was extracted using three different extraction protocols (see “DNA extraction protocols” below), and for each protocol a negative extraction (no sample) was included in duplicate. The DNA standard was taken along in duplicate. DNA was quantified using a Qubit 3.0 fluorometer (Invitrogen, Breda, The Netherlands) and the Qubit double-stranded DNA (dsDNA) HS assay kit (Thermo Fisher, Landsmeer, The Netherlands). A schematic overview of the study setup is shown in Fig. 1.

### DNA extraction protocols.

Detailed protocols, including all minor adaptations, are present in Text S1 in the supplemental material. DNA extraction was performed using three methods: (i) the Quick-DNA fecal/soil microbe kit (here referred to as Zymo) (Zymo Research) according to the manufacturer’s instructions with minor adaptations, (ii) protocol Q (here referred to as Q) (9), and (iii) automated DNA extraction with MagNA Pure 96 (here referred to as Magna) (Roche Diagnostics, Almere, The Netherlands) using the MagNA Pure 96 DNA and viral nucleic acid (NA) small-volume kit (Roche Diagnostics), according to standard operating procedures with minor adaptations. Mock communities were diluted to 10^4^ to 10^5^ cells per sample for extraction using Magna. For Q, several buffers and other materials were not provided in the kit and therefore were purchased elsewhere, namely, BeadBug prefilled tubes with 2.0-ml capacity and 0.1-mm zirconium beads (Sigma-Aldrich, Zwijndrecht, The Netherlands), RNase A, DNase and protease-free water (10 mg/ml) (Thermo Fisher, The Netherlands), and Tris-EDTA (TE) buffer (Thermo Fisher).

10.1128/mSystems.00547-19.1 TEXT S1 Detailed DNA extraction protocols. Download Text S1, PDF file, 0.3 MB. Copyright © 2020 Ducarmon et al. 2020 Ducarmon et al. This content is distributed under the terms of the Creative Commons Attribution 4.0 International license.

### MALDI-TOF mass spectrometry (Biotyper).

To verify whether all bacteria of the ATCC mock were lysed after the first mechanical lysis step of both Zymo and Q, the lysate was plated on a tryptic soy agar plate containing 5% sheep blood (VWR International, Amsterdam, The Netherlands), and aerobically and anaerobically incubated at 37°C for 5 days. The matrix-assisted laser desorption ionization–time of flight mass spectrometry (MALDI-TOF MS) Biotyper system was used (Bruker Daltonics, Germany) to identify the bacterial species. Samples were prepared in the following way. A bacterial colony was taken from the culturing plate and spread in duplicate on single spots on a Bruker polished steel target plate. Subsequently, 1 μl of 70% formic acid was added on each single spot, and when dried, 1 μl of prepared Bruker matrix α-cyano-4-hydroxycinnamic acid (HCCA) according to clinical laboratory protocols was added per spot. The Bruker polished steel target plate was then used for MALDI-TOF MS Biotyper analysis.

### Library preparation and 16S rRNA gene amplicon sequencing.

Of each duplicate DNA extraction from biological specimens, the duplicate with the highest genomic DNA concentration was used for sequencing. Duplicate samples from controls were both sequenced. Quality control, library preparation, and sequencing were performed by GenomeScan B.V. (Leiden, The Netherlands) using the NEXTflex 16S V4 Amplicon-Seq kit (BiooScientific, TX) and Illumina NextSeq 500 (paired end, 150 bp) according to their standard operating procedures. QC passing was based on intact genomic DNA and DNA concentrations measured by GenomeScan B.V. Therefore, those DNA concentrations were used for downstream analysis. Several samples were sequenced on multiple lanes, as indicated in all relevant figures and tables.

### Sequencing data analysis.

Read filtering, operational taxonomic unit (OTU) picking, and taxonomic assignment were performed using two different bioinformatic pipelines, QIIME 2 and NG-Tax 0.4 (49, 50), both using the Silva_132_SSU Ref database for taxonomic classification (51). For both pipelines, a read length of 120 bp was chosen based on quality of reads. The following settings were applied for QIIME 2: forward and reverse read length of 120 bp, quality control using Deblur, and identity level of 100% (default). The following settings were applied for NG-Tax: forward and reverse read length of 120 bp, ratio OTU abundance of 2.0 (default), classification ratio of 0.9 (default), minimum threshold of 0.1% (default), identity level of 100% (default), and error correction of 98.5 (default). Prior to the NG-Tax run, potential leftover primers were removed with cutadapt v.1.9.1 (52), in paired-end mode, with additional setting -e 0.2 (increased error tolerance, 20%). This setting was required since NG-Tax first creates a smaller custom database, based on the used primers. During further processing, data have to be primer sequence free, as the primer sequence is removed from the smaller database. Furthermore, all sequences with any deviating barcode in the fastq header were changed to the original barcode to allow inclusion into the NG-Tax pipeline.

The obtained OTU tables were filtered for OTUs with a number of sequences less than 0.005% of the total number of sequences (53). Downstream analysis was performed in R (v.3.6.1), mainly using the phyloseq (v.1.28.0), microbiome (v.1.6.0), and ggplot2 (v.3.2.0) packages (54–56). Alpha diversity was computed at both the OTU and genus levels, while analysis of compositional profiles was performed at the genus level. Kullback-Leibler divergence and Bray-Curtis dissimilarity measure heat maps were computed by first deleting genera that had a relative abundance of zero in all investigated samples (positive controls, feces, and oral swabs) and subsequent calculation of the respective measure. All R code is available upon request from the corresponding author.

### Data availability.

All raw sequencing data used in the current study are deposited in the European Nucleotide Archive under accession number PRJEB34118.

## References

[1] Guarner F, Malagelada JR 2003 Gut flora in health and disease. Lancet 361:512–519. doi:10.1016/S0140-6736(03)12489-0. 12583961

[2] Rajilic-Stojanovic M, Smidt H, de Vos WM 2007 Diversity of the human gastrointestinal tract microbiota revisited. Environ Microbiol 9:2125–2136. doi:10.1111/j.1462-2920.2007.01369.x. 17686012

[3] Human Microbiome Project Consortium. 2012 Structure, function and diversity of the healthy human microbiome. Nature 486:207–214. doi:10.1038/nature11234. 22699609 PMC3564958

[4] Sinha R, Abu-Ali G, Vogtmann E, Fodor AA, Ren B, Amir A, Schwager E, Crabtree J, Ma S, Microbiome Quality Control Project Consortium, Abnet CC, Knight R, White O, Huttenhower C 2017 Assessment of variation in microbial community amplicon sequencing by the Microbiome Quality Control (MBQC) project consortium. Nat Biotechnol 35:1077–1086. doi:10.1038/nbt.3981. 28967885 PMC5839636

[5] Fouhy F, Deane J, Rea MC, O’Sullivan Ó, Ross RP, O’Callaghan G, Plant BJ, Stanton C 2015 The effects of freezing on faecal microbiota as determined using MiSeq sequencing and culture-based investigations. PLoS One 10:e0119355. doi:10.1371/journal.pone.0119355. 25748176 PMC4352061

[6] Kennedy NA, UK IBD Genetics Consortium, Walker AW, Berry SH, Duncan SH, Farquarson FM, Louis P, Thomson JM, Consortium UIG, Satsangi J, Flint HJ, Parkhill J, Lees CW, Hold GL 2014 The impact of different DNA extraction kits and laboratories upon the assessment of human gut microbiota composition by 16S rRNA gene sequencing. PLoS One 9:e88982. doi:10.1371/journal.pone.0088982. 24586470 PMC3933346

[7] Walker AW, Martin JC, Scott P, Parkhill J, Flint HJ, Scott KP 2015 16S rRNA gene-based profiling of the human infant gut microbiota is strongly influenced by sample processing and PCR primer choice. Microbiome 3:26. doi:10.1186/s40168-015-0087-4. 26120470 PMC4482049

[8] Schirmer M, Ijaz UZ, D’Amore R, Hall N, Sloan WT, Quince C 2015 Insight into biases and sequencing errors for amplicon sequencing with the Illumina MiSeq platform. Nucleic Acids Res 43:e37. doi:10.1093/nar/gku1341. 25586220 PMC4381044

[9] Costea PI, Zeller G, Sunagawa S, Pelletier E, Alberti A, Levenez F, Tramontano M, Driessen M, Hercog R, Jung F-E, Kultima JR, Hayward MR, Coelho LP, Allen-Vercoe E, Bertrand L, Blaut M, Brown JRM, Carton T, Cools-Portier S, Daigneault M, Derrien M, Druesne A, de Vos WM, Finlay BB, Flint HJ, Guarner F, Hattori M, Heilig H, Luna RA, van Hylckama Vlieg J, Junick J, Klymiuk I, Langella P, Le Chatelier E, Mai V, Manichanh C, Martin JC, Mery C, Morita H, O’Toole PW, Orvain C, Patil KR, Penders J, Persson S, Pons N, Popova M, Salonen A, Saulnier D, Scott KP, Singh B, Slezak K, Veiga P, Versalovic J, Zhao L, Zoetendal EG, Ehrlich SD, Dore J, Bork P 2017 Towards standards for human fecal sample processing in metagenomic studies. Nat Biotechnol 35:1069–1076. doi:10.1038/nbt.3960. 28967887

[10] Santiago A, Panda S, Mengels G, Martinez X, Azpiroz F, Dore J, Guarner F, Manichanh C 2014 Processing faecal samples: a step forward for standards in microbial community analysis. BMC Microbiol 14:112. doi:10.1186/1471-2180-14-112. 24884524 PMC4021188

[11] Eisenhofer R, Minich JJ, Marotz C, Cooper A, Knight R, Weyrich LS 2019 Contamination in low microbial biomass microbiome studies: issues and recommendations. Trends Microbiol 27:105–117. doi:10.1016/j.tim.2018.11.003. 30497919

[12] Kuczynski J, Lauber CL, Walters WA, Parfrey LW, Clemente JC, Gevers D, Knight R 2011 Experimental and analytical tools for studying the human microbiome. Nat Rev Genet 13:47–58. doi:10.1038/nrg3129. 22179717 PMC5119550

[13] Zeeuwen P, Boekhorst J, Ederveen THA, Kleerebezem M, Schalkwijk J, van Hijum S, Timmerman HM 2017 Reply to Meisel et al. J Invest Dermatol 137:961–962. doi:10.1016/j.jid.2016.11.013. 27887953

[14] Jovel J, Patterson J, Wang W, Hotte N, O’Keefe S, Mitchel T, Perry T, Kao D, Mason AL, Madsen KL, Wong GK-S 2016 Characterization of the gut microbiome using 16S or shotgun metagenomics. Front Microbiol 7:459. doi:10.3389/fmicb.2016.00459. 27148170 PMC4837688

[15] Kullback S, Leibler RA 1951 On information and sufficiency. Ann Math Statist 22:79–86. doi:10.1214/aoms/1177729694.

[16] Hornung BVH, Zwittink RD, Kuijper EJ 2019 Issues and current standards of controls in microbiome research. FEMS Microbiol Ecol 95:fiz045. 30997495 10.1093/femsec/fiz045 PMC6469980

[17] Sun DL, Jiang X, Wu QL, Zhou NY 2013 Intragenomic heterogeneity of 16S rRNA genes causes overestimation of prokaryotic diversity. Appl Environ Microbiol 79:5962–5969. doi:10.1128/AEM.01282-13. 23872556 PMC3811346

[18] Patin NV, Kunin V, Lidstrom U, Ashby MN 2013 Effects of OTU clustering and PCR artifacts on microbial diversity estimates. Microb Ecol 65:709–719. doi:10.1007/s00248-012-0145-4. 23233090

[19] Scherz V, Aeby S, Bertelli C, Greub G 2019. Microbiota profiling of saliva: unexpected results of a preliminary study. Abstr ECCMID, Amsterdam, 2019.

[20] ATCC. 2018 Microbiome standard & research solutions. https://www.lgcstandards-atcc.org/~/media/A36E4E940E3F40B1A5E14D6851E9348F.ashx. Accessed 10 April 2019.

[21] Angelakis E, Bachar D, Henrissat B, Armougom F, Audoly G, Lagier JC, Robert C, Raoult D 2016 Glycans affect DNA extraction and induce substantial differences in gut metagenomic studies. Sci Rep 6:26276. doi:10.1038/srep26276. 27188959 PMC4870698

[22] Salter SJ, Cox MJ, Turek EM, Calus ST, Cookson WO, Moffatt MF, Turner P, Parkhill J, Loman NJ, Walker AW 2014 Reagent and laboratory contamination can critically impact sequence-based microbiome analyses. BMC Biol 12:87. doi:10.1186/s12915-014-0087-z. 25387460 PMC4228153

[23] Costello M, Fleharty M, Abreu J, Farjoun Y, Ferriera S, Holmes L, Granger B, Green L, Howd T, Mason T, Vicente G, Dasilva M, Brodeur W, DeSmet T, Dodge S, Lennon NJ, Gabriel S 2018 Characterization and remediation of sample index swaps by non-redundant dual indexing on massively parallel sequencing platforms. BMC Genomics 19:332. doi:10.1186/s12864-018-4703-0. 29739332 PMC5941783

[24] Minich JJ, Sanders JG, Amir A, Humphrey G, Gilbert JA, Knight R 2019 Quantifying and understanding well-to-well contamination in microbiome research. mSystems 4:e00186-19. doi:10.1128/mSystems.00186-19. PMC6593221 31239396

[25] Reigadas E, Vazquez-Cuesta S, Onori R, Villar-Gomara L, Alcala L, Marin M, Martin A, Munoz P, Bouza E 5 7 2019 Clostridioides difficile contamination in the environment of a clinical microbiology laboratory and laboratory workers. Clin Microbiol Infect doi:10.1016/j.cmi.2019.06.027. 31284033

[26] Qin J, MetaHIT Consortium, Li R, Raes J, Arumugam M, Burgdorf KS, Manichanh C, Nielsen T, Pons N, Levenez F, Yamada T, Mende DR, Li J, Xu J, Li S, Li D, Cao J, Wang B, Liang H, Zheng H, Xie Y, Tap J, Lepage P, Bertalan M, Batto JM, Hansen T, Le Paslier D, Linneberg A, Nielsen HB, Pelletier E, Renault P, Sicheritz-Ponten T, Turner K, Zhu H, Yu C, Li S, Jian M, Zhou Y, Li Y, Zhang X, Li S, Qin N, Yang H, Wang J, Brunak S, Dore J, Guarner F, Kristiansen K, Pedersen O, Parkhill J, Weissenbach J, Bork P, Ehrlich SD, Wang J 2010 A human gut microbial gene catalogue established by metagenomic sequencing. Nature 464:59–65. doi:10.1038/nature08821. 20203603 PMC3779803

[27] Lloyd-Price J, Abu-Ali G, Huttenhower C 2016 The healthy human microbiome. Genome Med 8:51. doi:10.1186/s13073-016-0307-y. 27122046 PMC4848870

[28] Lamont RJ, Koo H, Hajishengallis G 2018 The oral microbiota: dynamic communities and host interactions. Nat Rev Microbiol 16:745–759. doi:10.1038/s41579-018-0089-x. 30301974 PMC6278837

[29] Aas JA, Paster BJ, Stokes LN, Olsen I, Dewhirst FE 2005 Defining the normal bacterial flora of the oral cavity. J Clin Microbiol 43:5721–5732. doi:10.1128/JCM.43.11.5721-5732.2005. 16272510 PMC1287824

[30] Wolfe AJ, Toh E, Shibata N, Rong R, Kenton K, Fitzgerald M, Mueller ER, Schreckenberger P, Dong Q, Nelson DE, Brubaker L 2012 Evidence of uncultivated bacteria in the adult female bladder. J Clin Microbiol 50:1376–1383. doi:10.1128/JCM.05852-11. 22278835 PMC3318548

[31] Hilt EE, McKinley K, Pearce MM, Rosenfeld AB, Zilliox MJ, Mueller ER, Brubaker L, Gai X, Wolfe AJ, Schreckenberger PC 2014 Urine is not sterile: use of enhanced urine culture techniques to detect resident bacterial flora in the adult female bladder. J Clin Microbiol 52:871–876. doi:10.1128/JCM.02876-13. 24371246 PMC3957746

[32] Karstens L, Asquith M, Caruso V, Rosenbaum JT, Fair DA, Braun J, Gregory WT, Nardos R, McWeeney SK 2018 Community profiling of the urinary microbiota: considerations for low-biomass samples. Nat Rev Urol 15:735–749. doi:10.1038/s41585-018-0104-z. 30315209 PMC6352978

[33] Bučević Popović V, Šitum M, Chow C-ET, Chan LS, Roje B, Terzić J 2018 The urinary microbiome associated with bladder cancer. Sci Rep 8:12157. doi:10.1038/s41598-018-29054-w. 30108246 PMC6092344

[34] Boers SA, Hays JP, Jansen R 2017 Novel micelle PCR-based method for accurate, sensitive and quantitative microbiota profiling. Sci Rep 7:45536. doi:10.1038/srep45536. 28378789 PMC5381217

[35] Lim Y, Totsika M, Morrison M, Punyadeera C 2017 The saliva microbiome profiles are minimally affected by collection method or DNA extraction protocols. Sci Rep 7:8523. doi:10.1038/s41598-017-07885-3. 28819242 PMC5561025

[36] Vesty A, Biswas K, Taylor MW, Gear K, Douglas RG 2017 Evaluating the impact of DNA extraction method on the representation of human oral bacterial and fungal communities. PLoS One 12:e0169877. doi:10.1371/journal.pone.0169877. 28099455 PMC5242530

[37] Kostic AD, Chun E, Robertson L, Glickman JN, Gallini CA, Michaud M, Clancy TE, Chung DC, Lochhead P, Hold GL, El-Omar EM, Brenner D, Fuchs CS, Meyerson M, Garrett WS 2013 Fusobacterium nucleatum potentiates intestinal tumorigenesis and modulates the tumor-immune microenvironment. Cell Host Microbe 14:207–215. doi:10.1016/j.chom.2013.07.007. 23954159 PMC3772512

[38] He Z, Gharaibeh RZ, Newsome RC, Pope JL, Dougherty MW, Tomkovich S, Pons B, Mirey G, Vignard J, Hendrixson DR, Jobin C 2019 Campylobacter jejuni promotes colorectal tumorigenesis through the action of cytolethal distending toxin. Gut 68:289–300. doi:10.1136/gutjnl-2018-317200. 30377189 PMC6352414

[39] Kwong TNY, Wang X, Nakatsu G, Chow TC, Tipoe T, Dai RZW, Tsoi KKK, Wong MCS, Tse G, Chan MTV, Chan FKL, Ng SC, Wu JCY, Wu WKK, Yu J, Sung JJY, Wong SH 2018 Association between bacteremia from specific microbes and subsequent diagnosis of colorectal cancer. Gastroenterology 155:383–390.e8. doi:10.1053/j.gastro.2018.04.028. 29729257

[40] Abdulrahman Z, Kortekaas KE, De Vos Van Steenwijk PJ, Van Der Burg SH, Van Poelgeest MI 2018 The immune microenvironment in vulvar (pre)cancer: review of literature and implications for immunotherapy. Expert Opin Biol Ther 18:1223–1233. doi:10.1080/14712598.2018.1542426. 30373410

[41] van de Nieuwenhof HP, van der Avoort IA, de Hullu JA 2008 Review of squamous premalignant vulvar lesions. Crit Rev Oncol Hematol 68:131–156. doi:10.1016/j.critrevonc.2008.02.012. 18406622

[42] Vongsa R, Hoffman D, Shepard K, Koenig D 2019 Comparative study of vulva and abdominal skin microbiota of healthy females with high and average BMI. BMC Microbiol 19:16. doi:10.1186/s12866-019-1391-0. 30654751 PMC6337831

[43] Fuks G, Elgart M, Amir A, Zeisel A, Turnbaugh PJ, Soen Y, Shental N 2018 Combining 16S rRNA gene variable regions enables high-resolution microbial community profiling. Microbiome 6:17. doi:10.1186/s40168-017-0396-x. 29373999 PMC5787238

[44] van der Maaten L, Hinton G 2008 Visualizing data using t-SNE. Mach Learn 9:2579–2605. doi:10.1007/s10994-011-5273-4.

[45] Lauder AP, Roche AM, Sherrill-Mix S, Bailey A, Laughlin AL, Bittinger K, Leite R, Elovitz MA, Parry S, Bushman FD 2016 Comparison of placenta samples with contamination controls does not provide evidence for a distinct placenta microbiota. Microbiome 4:29. doi:10.1186/s40168-016-0172-3. 27338728 PMC4917942

[46] de Goffau MC, Lager S, Sovio U, Gaccioli F, Cook E, Peacock SJ, Parkhill J, Charnock-Jones DS, Smith G 2019 Human placenta has no microbiome but can contain potential pathogens. Nature 572:329–334. doi:10.1038/s41586-019-1628-y. 31367035 PMC6697540

[47] Reference deleted.

[48] Pushalkar S, Hundeyin M, Daley D, Zambirinis CP, Kurz E, Mishra A, Mohan N, Aykut B, Usyk M, Torres LE, Werba G, Zhang K, Guo Y, Li Q, Akkad N, Lall S, Wadowski B, Gutierrez J, Kochen Rossi JA, Herzog JW, Diskin B, Torres-Hernandez A, Leinwand J, Wang W, Taunk PS, Savadkar S, Janal M, Saxena A, Li X, Cohen D, Sartor RB, Saxena D, Miller G 2018 The pancreatic cancer microbiome promotes oncogenesis by induction of innate and adaptive immune suppression. Cancer Discov 8:403–416. doi:10.1158/2159-8290.CD-17-1134. 29567829 PMC6225783

[49] Ramiro-Garcia J, Hermes GDA, Giatsis C, Sipkema D, Zoetendal EG, Schaap PJ, Smidt H 2016 NG-Tax, a highly accurate and validated pipeline for analysis of 16S rRNA amplicons from complex biomes [version 1; referees: 2 approved with reservations]. F1000Res 5:1791. doi:10.12688/f1000research.9227.1. 30918626 PMC6419982

[50] Bolyen E, Rideout JR, Dillon MR, Bokulich NA, Abnet C, Al-Ghalith GA, Alexander H, Alm EJ, Arumugam M, Asnicar F, Bai Y, Bisanz JE, Bittinger K, Brejnrod A, Brislawn CJ, Brown CT, Callahan BJ, Caraballo-Rodríguez AM, Chase J, Cope E, Da Silva R, Dorrestein PC, Douglas GM, Durall DM, Duvallet C, Edwardson CF, Ernst M, Estaki M, Fouquier J, Gauglitz JM, Gibson DL, Gonzalez A, Gorlick K, Guo J, Hillmann B, Holmes S, Holste H, Huttenhower C, Huttley G, Janssen S, Jarmusch AK, Jiang L, Kaehler B, Kang KB, Keefe CR, Keim P, Kelley ST, Knights D, Koester I, Kosciolek T, Kreps J, Langille MGI, Lee J, Ley R, Liu Y-X, Loftfield E, Lozupone C, Maher M, Marotz C, Martin BD, McDonald D, McIver LJ, Melnik AV, Metcalf JL, Morgan SC, Morton J, Naimey AT, Navas-Molina JA, Nothias LF, Orchanian SB, Pearson T, Peoples SL, Petras D, Preuss ML, Pruesse E, Rasmussen LB, Rivers A, Robeson I, Rosenthal P, Segata N, Shaffer M, Shiffer A, Sinha R, Song SJ, Spear JR, Swafford AD, Thompson LR, Torres PJ, Trinh P, Tripathi A, Turnbaugh PJ, Ul-Hasan S, van der Hooft JJJ, Vargas F, Vázquez-Baeza Y, Vogtmann E, von Hippel M, Walters W, Wan Y, Wang M, Warren J, Weber KC, Williamson CHD, Willis AD, Xu ZZ, Zaneveld JR, Zhang Y, Zhu Q, Knight R, Caporaso JG 2018 QIIME 2: reproducible, interactive, scalable, and extensible microbiome data science. PeerJ Preprints 6:e27295v2. 10.1038/s41587-019-0209-9 PMC7015180 31341288

[51] Quast C, Pruesse E, Yilmaz P, Gerken J, Schweer T, Yarza P, Peplies J, Glockner FO 2013 The SILVA ribosomal RNA gene database project: improved data processing and web-based tools. Nucleic Acids Res 41:D590–D596. doi:10.1093/nar/gks1219. 23193283 PMC3531112

[52] Martin M 2011 Cutadapt removes adapter sequences from high-throughput sequencing reads. EMBnet J 17:3.

[53] Bokulich NA, Subramanian S, Faith JJ, Gevers D, Gordon JI, Knight R, Mills DA, Caporaso JG 2013 Quality-filtering vastly improves diversity estimates from Illumina amplicon sequencing. Nat Methods 10:57–59. doi:10.1038/nmeth.2276. 23202435 PMC3531572

[54] McMurdie PJ, Holmes S 2013 phyloseq: an R package for reproducible interactive analysis and graphics of microbiome census data. PLoS One 8:e61217. doi:10.1371/journal.pone.0061217. 23630581 PMC3632530

[55] Lahti L, Shetty S 2017 Tools for microbiome analysis in R. Microbiome package version 1.2.1.

[56] Wickham H 2009 ggplot2: elegant graphics for data analysis. Springer Science+Business Media, Berlin, Germany.

