# Supplementary figures and images for: Toward Standards in Clinical Microbiota Studies: Comparison of Three DNA Extraction Methods and Two Bioinformatic Pipelines
Source: mSystems. 2020 Feb 11;5(1):e00547-19. doi: 10.1128/mSystems.00547-19 (PMC7018525; doi:10.1128/mSystems.00547-19)

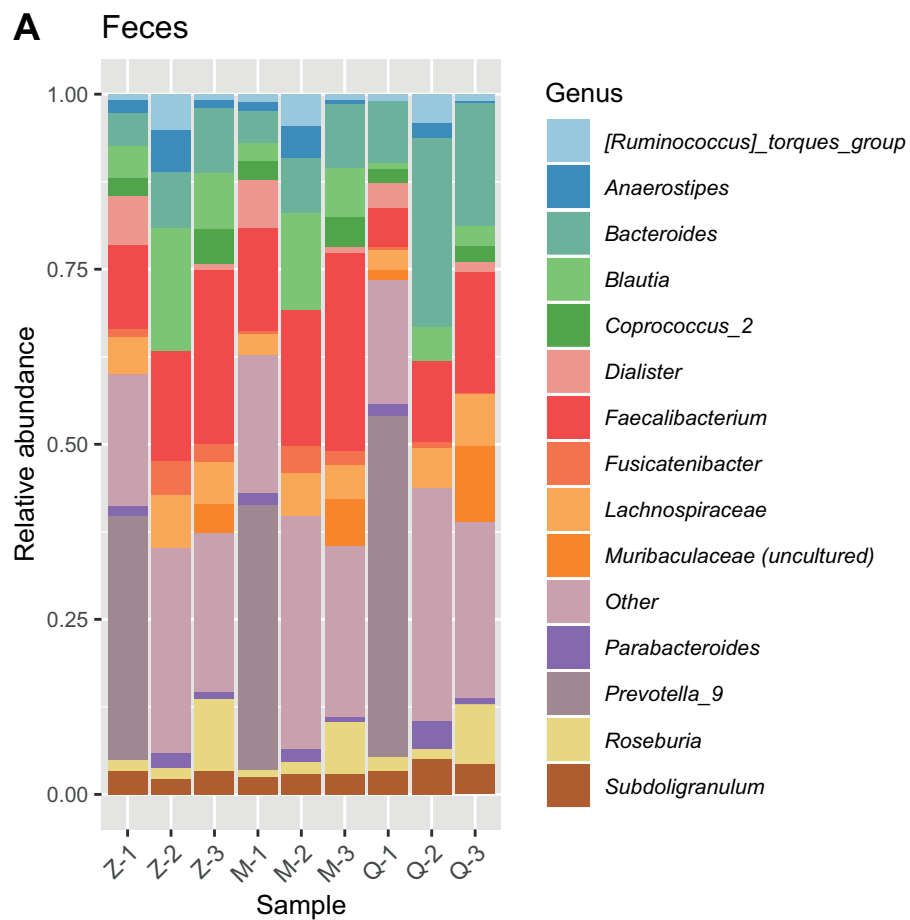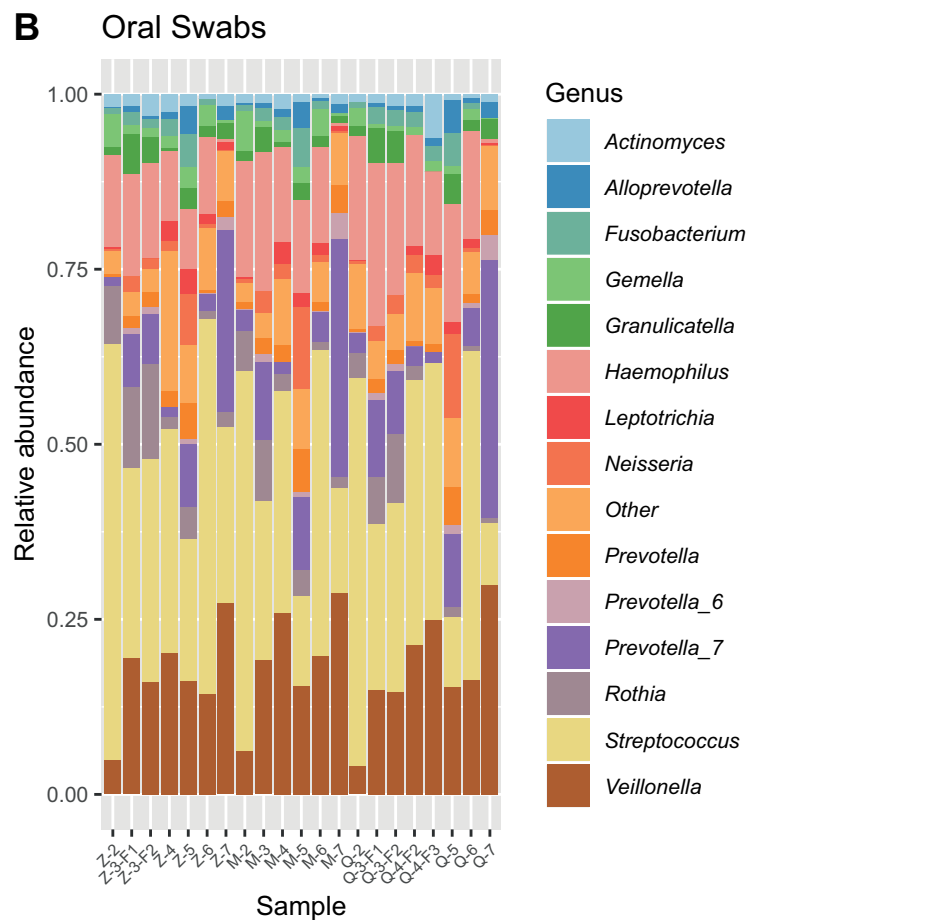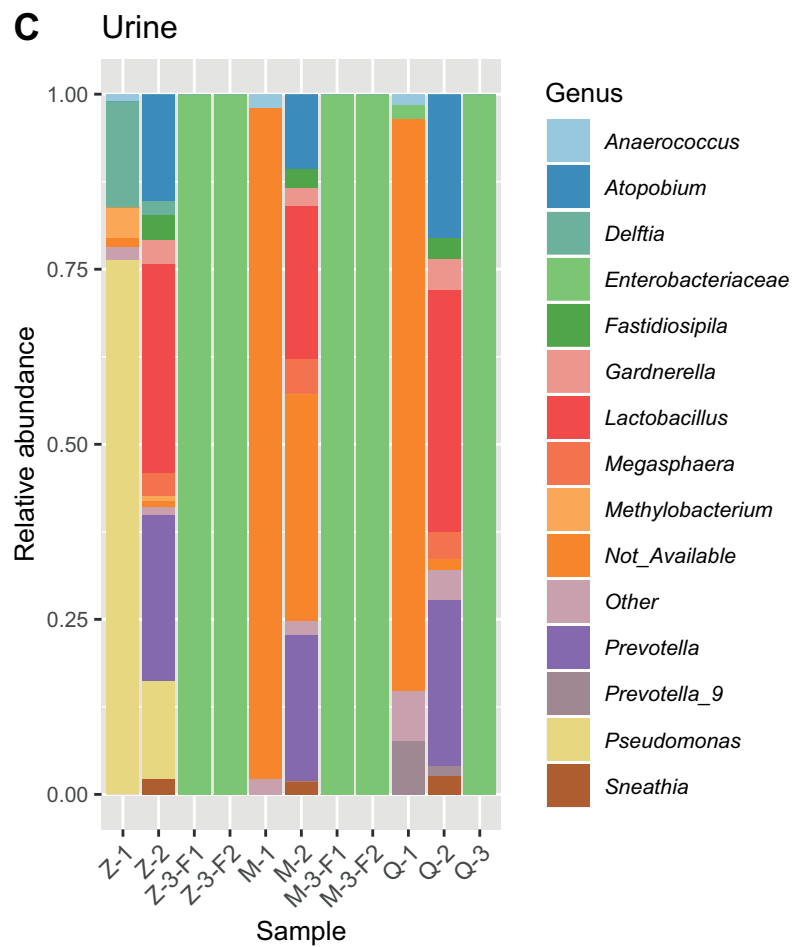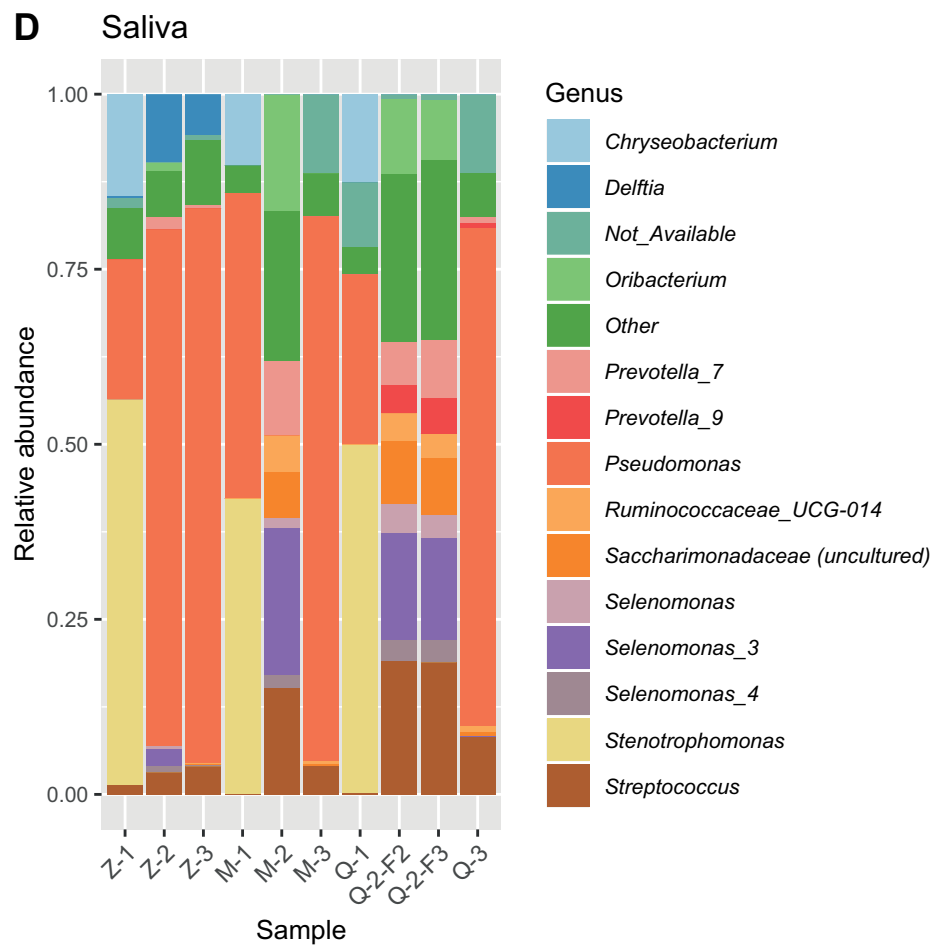

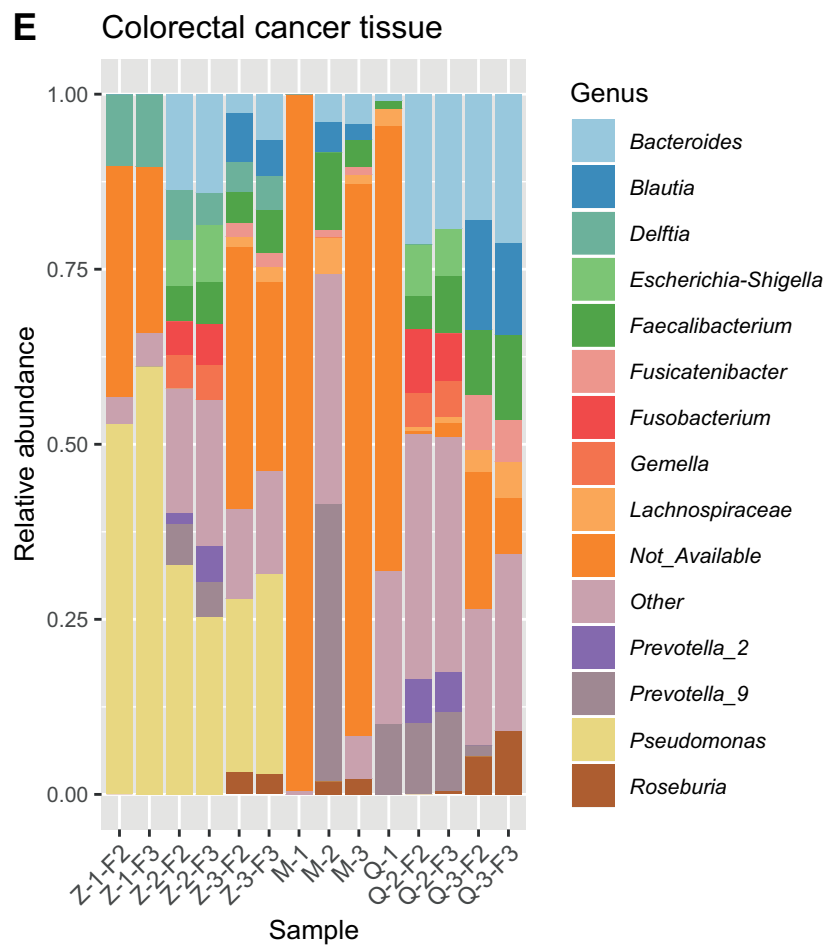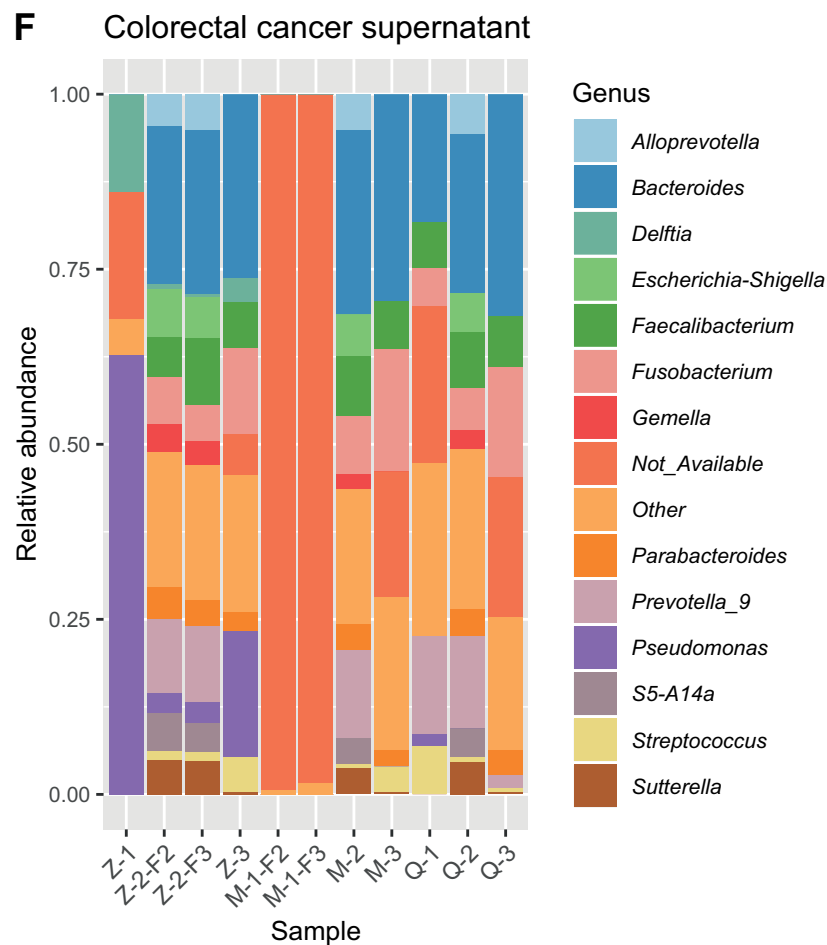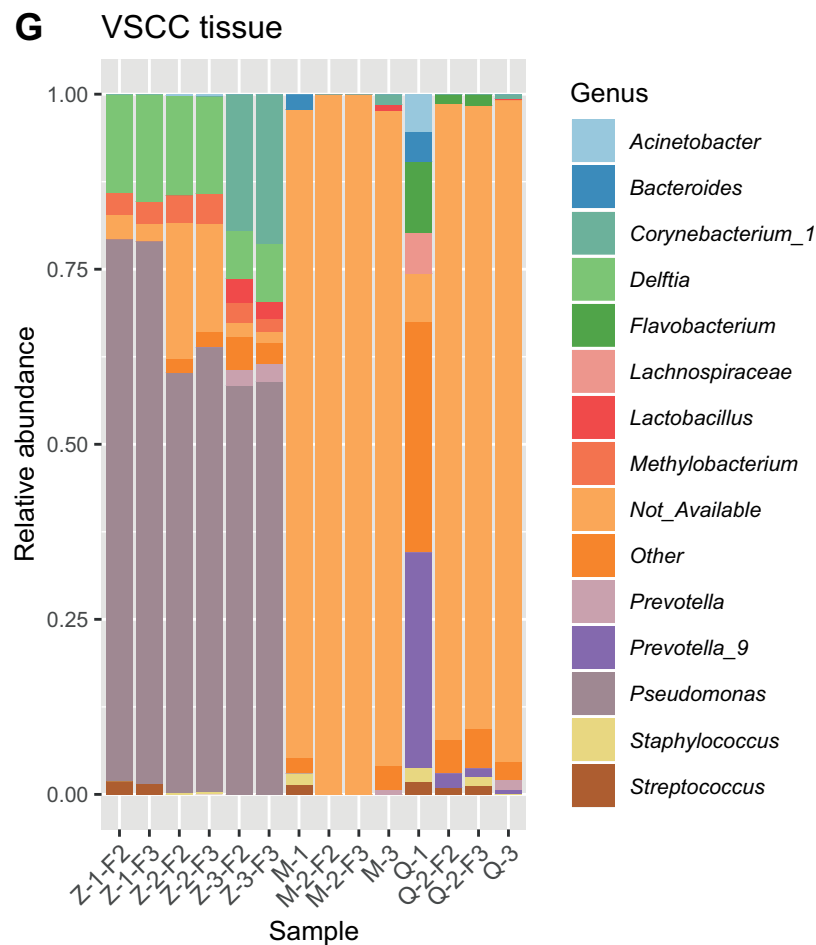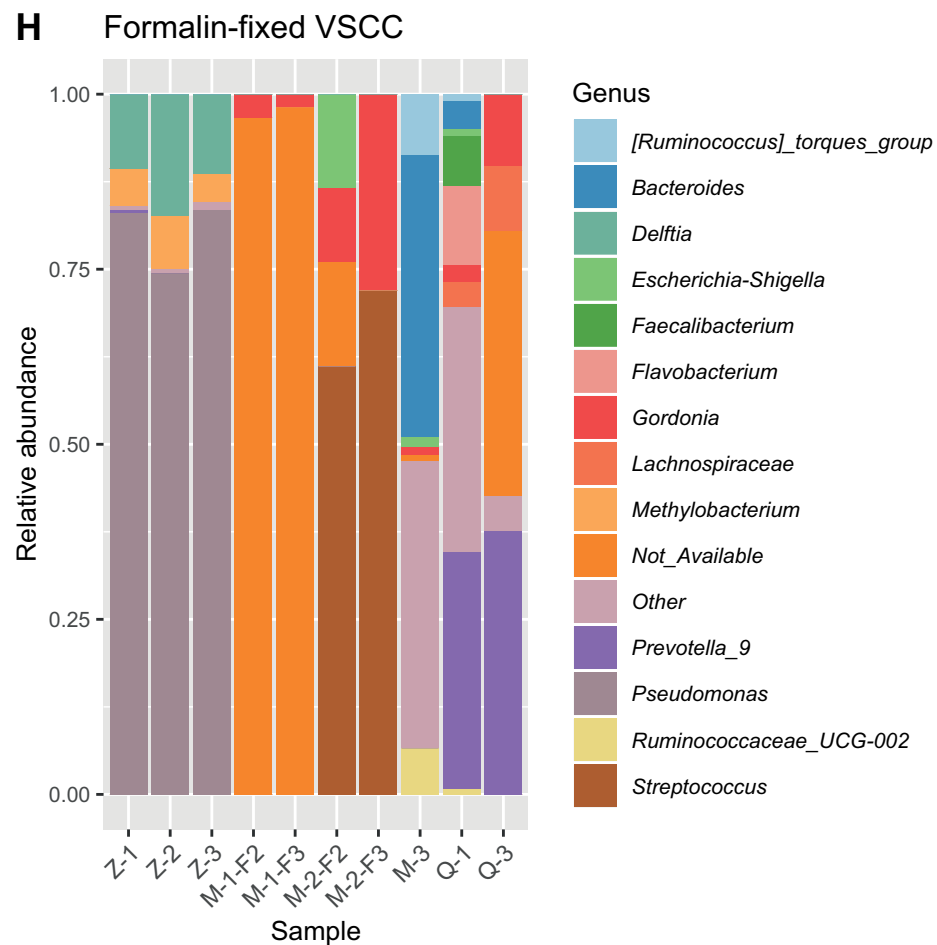

Supplement: FIG S3 [file mSystems.00547-19-sf003.pdf]
