# Supplementary material for: Toward Standards in Clinical Microbiota Studies: Comparison of Three DNA Extraction Methods and Two Bioinformatic Pipelines
Source: mSystems. 2020 Feb 11;5(1):e00547-19. doi: 10.1128/mSystems.00547-19 (PMC7018525; doi:10.1128/mSystems.00547-19)
Supplement: TEXT S1 [file mSystems.00547-19-s0001.pdf]

Supplementary methods

Toward standards in clinical microbiome studies: comparison of three DNA extraction methods and two bioinformatic pipelines

Q.R. Ducarmon[a, b], B.V.H. Hornung[a,b], A.R. Geelen[a,b], E.J. Kuijper[a,b], R.D. Zwittink[a,b]

[a]Center for Microbiome Analyses and Therapeutics, Leiden University Medical Center, Leiden, The Netherlands

[b]Department of Medical Microbiology, Leiden University Medical Center, Leiden, The Netherlands

Supplementary methods extraction protocols

Adaptations to the original extraction protocols are indicated in red

Zymo extraction protocol with minor adaptations

1. Add ≤ 150 mg of fecal sample or ≤ 250 mg of soil sample to a ZR BashingBead™ Lysis Tube (0.1 & 0.5 mm). Add 750 µl BashingBead™ Buffer to the tube1 . Note: Alternatively, add water sampl2 or 50-100 mg (wet weight) fungal/bacterial cells that have been resuspended in up to 200 µl of water or isotonic buffer (e.g., PBS) to a ZR BashingBead™ Lysis Tube.

2. Secure in a Qiagen Tissue Lyser LT fitted with a 2 ml tube holder assembly and process at 50Hz for 10 minutes. Note: Required processing time will vary depending on the device and application and therefore should be evaluated on a case by case basis. For example, processing times may be as little as 3 minutes when using high-speed cell disrupters (e.g., the portable TerraLyzer™ Sample Processor, FastPrep® -24, or similar) or as long as 20 minutes when using lower speeds (e.g., Disruptor Genie™, or standard benchtop vortexes). See manufacturer's literature for operating information.

3. Centrifuge the ZR BashingBead™ Lysis Tube (0.1 & 0.5 mm) in a microcentrifuge at ≥ 10,000 x g for 1 minute.

4. Transfer up to 400 µl supernatant to a Zymo-Spin™ III-F Filter in a Collection Tube and centrifuge at 8,000 x g for 1 minute.

5. Binding preparation: Feces and All Non-Soil Samples. Add 1,200 µl of Genomic Lysis Buffer to the filtrate in the Collection Tube from Step 4. Mix wel. Mix well.

6. Transfer 800 µl of the mixture from Step 5 to a Zymo-Spin™ IIC Column4 in a Collection Tube and centrifuge at 10,000 x g for 1 minute.

7. Discard the flow through from the Collection Tube and repeat Step 6.

8. Add 200 µl DNA Pre-Wash Buffer to the Zymo-Spin™ IIC Column in a new Collection Tube and centrifuge at 10,000 x g for 1 minute.

9. Add 500 µl g-DNA Wash Buffer to the Zymo-Spin™ IIC Column and centrifuge at 10,000 x g for 1 minute.

10. Transfer the Zymo-Spin™ IIC Column to a clean 1.5 ml microcentrifuge tube and add 100 µl (50 µl minimum) DNA Elution Buffer directly to the column matrix. Centrifuge at 10,000 x g for 30 seconds to elute the DNA and let the filtered eluate run through the column once more.

11. Place a Zymo-Spin™ III-HRC Filter in a clean Collection Tube and add 600 µl Prep Solution. Centrifuge at 8,000 x g for 3 minutes.

12. Transfer the eluted DNA to a prepared Zymo-Spin™ III-HRC Filter in a clean 1.5 ml microcentrifuge tube and centrifuge at exactly 16,000 x g for 3 minutes. The filtered DNA is now suitable for PCR and other downstream applications.

IHMS DNA extraction protocol Q Fecal DNA extraction with the use of Qiagen QIAamp DNA stool kit

1. Homogenize the 150 to 200mg frozen feces with 1.0mL ASL lysis buffer of the kit by vortexing for 2min in a 2mL tube containing 0.3g of sterile zirconia beads Ø 0,1mm zirconia (BioSpec, Cat. No. 11079101z). [if buffer shows precipitate, heat at 70°C before use]

2. Incubate for 15min at 95°C.

3. Cells are mechanically lysed by running the Qiagen Tissue Lyser LT for 10 minutes at 50Hz.

4. Samples are allowed to cool down on ice for 2min.

5. Samples are centrifuged at 16000 x g, 4°C, for 5min.

6. Supernatant is transferred to a new 2mL tube.

7. The pellet is mixed with 300µL ASL lysis buffer of the kit, and steps 2-5 are repeated.

8. Supernatants are pooled in the new 2mL tube.

9. Add 260µl of 10M ammonium acetate to each lysate tube, mix well, and incubate on ice for 5 min.

10. Centrifuge at 16000 g, 4°C, for 10min.

11. Transfer the supernatant to two 1.5mL Eppendorf tubes, add one volume of isopropanol, mix well, and incubate on ice for 30 min.

12. Centrifuge at 16000 g, 4°C, 15min, remove the supernatant using aspiration, wash nucleic acids pellet with 70 % EtOH (0,5mL) and dry the pellet under vacuum for 3min.

13. Dissolve the nucleic acid pellet in 100µL of TE (Tris-EDTA) buffer and pool the two aliquots.

14. Add 2µL of DNase-free RNase (10mg/mL) and incubate at 37°C, 15 min.

15. Add 15µL proteinase K and 200µL AL buffer to the supernatant, vortex for 15sec and incubate at 70°C for 10 min.

16. Add 200µL of ethanol (96-100%) to the lysate, and mix by vortexing.

17. Transfer to a QIAamp spin column and centrifuge at 16000 g for 1min, at Room Temperature (RT).

18. Discard flow through, add 500µL buffer AW1 (Qiagen) and centrifuge at 16000 g for 1min, at RT.

19. Discard flow through, add 500µL buffer AW2 (Qiagen) and centrifuge at 16000 g for 1min, at RT

20. Dry the column by centrifugation at RT for 1min. 21. Add 200µL Buffer AE (Qiagen), incubate for 1min at RT 22. Centrifuge for 1min at 16000 g to elute DNA.

MagNA Pure 96 ™ extraction protocol

1. Add maximum of 200 µl of each sample to 1 ml STAR buffer and Precellys beads, shortly vortex afterwards.

2. Bead-beat for 10 minutes, 50 Hz on Qiagen Tissue Lyser LT

3. Incubate for 5 minutes at room temperature, followed by 1 minute centrifugation on 14 000 rpm

4. Pipet 200 µl per sample in MagNA Pure 96 ™ processing cartridge for automated extraction using the Pathogen Universal Protocol 200.

5. Cartridge was used for DNA extraction by MagNA Pure 96 ™
